# First Experience of Three Neurovascular Centers With the p64MW-HPC, a Low-Profile Flow Diverter Designed for Proximal Cerebral Vessels With Antithrombotic Coating

**DOI:** 10.3389/fneur.2021.724705

**Published:** 2021-09-14

**Authors:** Helge Winters, Marie-Sophie Schüngel, Cordula Scherlach, Dirk Mucha, Jörg Thalwitzer, Wolfgang Härtig, Aneta Donitza, Nikolaos Bailis, Jens Maybaum, Karl Titus Hoffmann, Ulf Quäschling, Stefan Schob

**Affiliations:** ^1^Institut für Neuroradiologie, Universitätsklinikum Leipzig, Leipzig, Germany; ^2^Klinik und Poliklinik für Anästhesiologie und Intensivtherapie, Universitätsklinikum Leipzig, Leipzig, Germany; ^3^Institut für Radiologie und Neuroradiologie, Heinrich-Braun- Klinikum, Zwickau, Germany; ^4^Institut für Radiologie und Neuroradiologie, Klinikum Chemnitz gGmbH, Chemnitz, Germany; ^5^Paul-Flechsig-Institut für Hirnforschung, Universität Leipzig, Leipzig, Germany; ^6^Abteilung für Neuroradiologie, Klinik & Poliklinik für Radiologie, Universitätsklinikum Halle, Halle (Saale), Germany

**Keywords:** flow diverter, p64MW-HPC, hydrophilic coating, HPC, navigability, anti-platelet therapy

## Abstract

**Background:** In the last decade, flow diversion (FD) has been established as hemodynamic treatment for cerebral aneurysms arising from proximal and distal cerebral arteries. However, two significant limitations remain—the need for 0.027” microcatheters required for delivery of most flow diverting stents (FDS), and long-term dual anti-platelet therapy (DAPT) in order to prevent FDS-associated thromboembolism, at the cost of increasing the risk for hemorrhage. This study reports the experience of three neurovascular centers with the p64MW-HPC, a FDS with anti-thrombotic coating that is implantable *via* a 0.021” microcatheter.

**Materials and methods:** Three neurovascular centers contributed to this retrospective analysis of patients that had been treated with the p64MW-HPC between March 2020 and March 2021. Clinical data, aneurysm characteristics, and follow-up results, including procedural and post-procedural complications, were recorded. The hemodynamic effect was assessed using the O'Kelly–Marotta Scale (OKM).

**Results:** Thirty-two patients (22 female, mean age 57.1 years) with 33 aneurysms (27 anterior circulation and six posterior circulation) were successfully treated with the p64MW-HPC. In 30/32 patients (93.75%), aneurysmal perfusion was significantly reduced immediately post implantation. Follow-up imaging was available for 23 aneurysms. Delayed aneurysm perfusion (OKM A3: 8.7%), reduction in aneurysm size (OKM B1-3: 26.1%), or sufficient separation from the parent vessel (OKM C1-3 and D1: 65.2%) was demonstrated at the last available follow-up after a mean of 5.9 months. In two cases, device thrombosis after early discontinuation of DAPT occurred. One delayed rupture caused a caroticocavernous fistula. The complications were treated sufficiently and all patients recovered without permanent significant morbidity.

**Conclusion:** Treatment with the p64MW-HPC is safe and feasible and achieves good early aneurysm occlusion rates in the proximal intracranial circulation, which are comparable to those of well-established FDS. Sudden interruption of DAPT in the early post-interventional phase can cause in-stent thrombosis despite the HPC surface modification. Deliverability *via* the 0.021” microcatheter facilitates treatment in challenging vascular anatomies.

## Introduction

In the past decade, flow diversion (FD) has been well-established as a functional treatment option for cerebral aneurysms. The distinct architecture of fine-meshed flow diverter stents (FDS), characterized by an optimized ratio of porosity and surface area metal coverage, allows maintained perfusion of covered arteries while decreasing aneurysmal perfusion below the threshold required for intra-aneurysmal thrombosis (1). Following the immediate reduction of aneurysmal inflow with subsequent thrombosis, the FDS serves as scaffolding for the development of an increasingly resilient neo-intima (2–4).

Both events, flow reduction and formation of a neo-intima, depend on FDS porosity (5). Regarding the latter, computational fluid dynamics revealed that porosities of 70% or less are sufficient to trigger occlusion (6). Definite reconstruction of the aneurysm-bearing segment depends on the ingrowth of smooth muscle cells together with endothelial cells along the FDS at the level of the aneurysmal orifice, according to histological findings (7). The process of vascular remodeling starts at the peripheral landing zones of the FDS, continues inwardly, and strongly depends on sufficient wall apposition of the implant (7). In this study, completely occluded aneurysms were covered entirely with a thin but continuous layer of smooth muscle and endothelium at the neck level, whereas substantial aneurysmal remnants were associated with discontinued islands of inflammatory cells, mostly monocytes and macrophages, incompletely covering the FDS struts at the aneurysm neck. As a consequence, the immediate flow diverting effect of FDS should be considered only as a prerequisite for the proper therapeutic mechanism of FD, which is the establishment of a novel functional vessel wall that results from the complex interaction between a variety of cell types and the metal surface of the stent (8). Neo-endothelialization is initiated by activated platelets that bind to surface adsorbed fibrinogen *via* GPIIb/IIIa (9, 10). Those platelets not only mediate the recruitment of circulating endothelial progenitor cells to the FDS' surface and strongly support their proliferation and differentiation to functioning endothelial cells (11), but also trigger and further amplify local coagulation (12). Therefore, FD requires concomitant dual anti-platelet therapy (DAPT) to prevent thromboembolic complications, which are responsible for stroke with permanent neurological impairment in up to 7.4% of cases (13). However, DAPT reduces the risk for thromboembolic events at the cost of increasing the risk for hemorrhagic complications (14), and both hemorrhagic and ischemic events remain a significant concern in clinical practice, especially in ruptured aneurysms (15). Therefore, the ability to implant FDS in cerebral vessels without the imperative for DAPT would be a major improvement (10).

The p64MW-HPC (phenox, Bochum, Germany) is a novel FDS designed for cerebral vessels with diameters between 3.0 and 5.0 mm and can be implanted with 0.021” microcatheters. The device was developed with a special focus on enhanced hemocompatibility, which is achieved by a glycan-based, multilayer hydrophilic polymer coating (HPC) that inhibits the initial step of platelet adhesion to surface adsorbed fibrinogen (16). *Ex vivo* testing has demonstrated that platelets in contact with HPC-covered FDS are significantly less activated than platelets in contact with uncoated FDS (16). Additionally, first clinical evidence indicates that applying the HPC technology to FDS allows reduction of DAPT in selected cases of unruptured and even ruptured cerebral aneurysms (17–20). Whether the HPC technology may delay neo-endothelialization, and thus aneurysm occlusion, remains to be elucidated. As a consequence, this study aims to report the early experiences with the p64MW-HPC from three German neurovascular centers (University Hospital Leipzig, Heinrich-Braun-Hospital Zwickau, and Klinikum Chemnitz GmbH), specifically focusing on technical issues, thromboembolic or hemorrhagic complications, and aneurysm occlusion rates.

## Materials and Methods

### Ethics Approval

This retrospective study was approved by the institutional ethics committee (local IRB no. AZ 208-15-0010062015). Written consent was obtained from the patient or his or her legal representative.

### Study Design

The study was designed as a multi-center, single-arm retrospective analysis of a prospectively maintained database comprising all endovascular treatments with the p64MW-HPC performed in the following neurovascular centers: University Hospital Leipzig (*n* = 27), Heinrich-Braun-Hospital Zwickau (*n* = 3), and Klinikum Chemnitz (*n* = 2). Demographic data, aneurysm characteristics, procedural aspects, technical and clinical adverse events, as well as early angiographic follow-up results were systematically reviewed for all cases. Table 1 provides an overview of the ascertained data from all three neurovascular centers.

**Table 1:** Demographic data.

**Patient**	**Age**	**Sex**	**Location**	**Aneurysm configuration**	**Neck width (mm)**	**Dome width (mm)**	**Dome height (mm)**	**Treatment strategy**	**Antiplatelet therapy**	**Device**	**Size**	**OKM after FD**	**1st FU**	**OKM 1st FU**	**Last available FU**	**OKM last FU**
1	64	Female	Right C6/ C7	Saccular	2	4.7	3.3	Primary	ASA + Ticagrelor	1 × p64MW-HPC	3.5 × 18	B3	3 months	B3	3 months	B3
2	60	Female	Right M1	Saccular	3.3	4.2	3.3	Primary	ASA + Ticagrelor	1 × p64MW-HPC	3.5 × 18	A2	2.5 months	B2	10 months	B2
3	34	Male	Left C6	Saccular	4.5	4.7	4.3	Primary	ASA + Ticagrelor	1 × p64MW-HPC	5 × 30	B3	3.5 months	D1	12 months	D1
4	74	Female	Right C6	Saccular	3.7	4.3	2.7	Primary	ASA + Ticagrelor^*^	1 × p64MW-HPC	4.5 × 27	B3	1.5 weeks	B3	11 months	B3
5	27	Male	Left C6	Saccular	2.7	3.3	4	Primary	ASA + Ticagrelor	1 × p64MW-HPC	4 × 18	B2	3.5 months	B3	10 months	B2
6	65	Female	Left C6/ C7	Saccular	3.5	3.8	4.2	Primary	ASA + Clopidogrel	1 × p64MW-HPC	5 × 30	A1	3.5 months	A3	3.5 months	A3
7	49	Male	Right C7	Saccular (2×)	3 2.8	3.5 2.8	4.8 3.2	Primary Primary	ASA + Ticagrelor	1 × p64MW-HPC + Coil	4.5 × 15	A3 A3	4 months	D1 C2	10 months	D1 C2
8	56	Female	Right C4	Saccular	4.5	5.3	3.5	Primary	ASA + Ticagrelor	2 × p64MW-HPC	4 × 15 4.5 × 18	A3	3 months	D1	6.5 months	D1
9	55	Female	Basilar artery	Saccular	6.3	18	50	Revision	ASA + Ticagrelor + De × amethason	1 × p64MW-HPC	3.5 × 21	A3	4 months	B3	9 months	B3 (but distinctly decreased in aneurysm size)
10	63	Female	Left C4/ C5	Saccular	2.1	3.1	5.4	Primary	Ticagrelor	1 × p64MW-HPC	4 × 21	A2	4 months	C1	4 months	C1
11	36	Male	Right C7	Blister	-	-	-	Primary (acute SAH)	ASA + Ticagrelor	1 × p64MW-HPC	4 × 21	C2	1.5 weeks	D1	9 months	D1
12	68	Female	Left C6	Saccular	2.9	3.2	4.5	Primary	ASA + Ticagrelor	1 × p64MW-HPC	4 × 15	A3	3.5 months	C3	7 months	D1
13	72	Female	Right C3/ C4	Saccular	4	8	9	Primary	ASA + Ticagrelor	1 × p64MW-HPC	4 × 21	A3	3 weeks	A1	4 months	C2
14	72	Female	Right C3/ C4	Barrow A fistula	-	-	-	Revision	ASA + Ticagrelor	2 × p64MW-HPC + venous coiling	4 × 18 4 × 21	A1	5 days	A1	3 months	C2
15	58	Male	Right C6	Saccular	4	4.2	3.6	Primary	ASA + Ticagrelor	1 × p64MW-HPC	4.5 × 18	B3	2 months	C3	5.5 months	D1
16	62	Male	Left C4	Stenosis	-	-	-	Primary	ASA + Ticagrelor	2 × p64MW-HPC	3.5 × 12 3.5 × 21	n.a.	n.a.	n.a.	n.a.	n.a.
17	56	Female	Left V4/ PICA orifice	Saccular	3	3.6	5.6	Primary	Prasugrel ^**^	1 × p64MW-HPC	3 × 12	A3	3 months	D1	3 months	D1
18	68	Female	Left C3/C4	Saccular	8.4	14.2	14.3	Primary	Prasugrel ^**^	1 × p64MW-HPC + 2 × PED2	5 × 30	A3	3.5 months	A3	3.5 months	A3
19	64	Female	Right C6/ C7, Pcom orifice	Saccular	5.2	6.3	8.2	Primary	ASA + Ticagrelor	1 × p64MW-HPC	5 × 30	B3	3 months	B2	3 months	B2
20	48	Female	Right C4	Saccular	3.2	5.2	4.3	Revision	ASA + Ticagrelor	2 × p64MW-HPC	3.5 × 15 3.5 × 18	A3	n.a.	n.a.	n.a.	n.a.
21	48	Male	Left C6	Saccular	5	26	17	Primary	Prasugrel + Celebre × + De × amethason	1 × p64MW-HPC + Coiling	4.5 × 21	B3	3 months	C2	3 months	C2
22	24	Female	Right C6	Saccular	2.9	3.5	3.3	Primary	ASA + Prasugrel	1 × p64MW-HPC	3.5 × 18	A2	3 months	D1	3 months	D1
23	60	Male	Right C6, AChA orifice	Saccular	1	1.8	2.9	Revision	ASA + Prasugrel	1 × p64MW-HPC	3.5 × 15	B2	n.a.	n.a.	n.a.	n.a.
24	68	Female	Right C6	Saccular	2.6	3.4	3.8	Primary	ASA + Ticagrelor	1 × p64MW-HPC	3.5 × 18	A2	n.a.	n.a.	n.a.	n.a.
25	50	Female	Left V4/ PICA orifice	Saccular	2.8	5	4	Primary	ASA + Prasugrel	1 × p64MW-HPC	4 × 12	A2	n.a.	n.a.	n.a.	n.a.
26	46	Female	Right C6	Saccular	2.3	3	4.3	Primary	ASA + Ticagrelor	1 × p64MW-HPC + 1 × SVB	3 × 15	A3	n.a.	n.a.	n.a.	n.a.
27	82	Female	Right C7	Saccular	4.5	4.5	5	Primary	ASA + Clopidogrel ^**^	1 × p64MW-HPC	3.5 × 18	A3	n.a.	n.a.	n.a.	n.a.
28	43	Female	Basilar artery tip	Saccular	7	5	5	Primary	ASA + Ticagrelor	1 × p64MW-HPC	3.5 × 21	A2	n.a.	n.a.	n.a.	n.a.
29	58	Female	Left V4	Dissection	-	-	-	Primary (acute SAH)	ASA + Ticagrelor	1 × p64MW-HPC	3 × 18	D1	n.a.	n.a.	n.a.	n.a.
30	52	Male	Left C6, Pcom orifice	Saccular	4	4	5	Primary	ASA + Clopidogrel	1 × p64MW-HPC	4 × 15	A2	n.a.	n.a.	n.a.	n.a.
31	75	Female	Left C6, AChA orifice	Saccular	4	4	3	Primary	ASA + Prasugrel	2 × p64MW-HPC	4 × 18 4 × 21	A2	1 week	A2	6 months	C2
32	71	Male	Basilar artery/ left V4	Fusiform	20	10	10	Primary	ASA + Clopidogrel	3 × p64MW-HPC	4.5 × 27 5 × 24 5 × 30	A2	1 week	C1	1 week	C1

* *Two months after flow diversion, the DAPT was changed to ASA + Prasugrel due to recurrent events of inadaequate administration of ticagrelor*.

** *In addition, oral anticoagulation was administered due to cardiologic indication*.

### The p64MW-HPC—Features

The p64MW-HPC has been designed and approved for the therapy of cerebral aneurysms arising from vessels with 3.0–5.0 mm in diameter. The device consists of 64 braided wires, composed of nickel–titanium alloy with an inner platinum core. Contrary to the majority of other FDS, which were approved for similarly sized cerebral vessels but require a 0.027” microcatheter for delivery (for example: Surpass—Stryker Neurovascular, FRED—Microvention, Pipeline Embolization Device—Medtronic, Derivo—Acandis), the p64MW-HPC only requires a 0.021” microcatheter. The smaller microcatheter allows enhanced access and less traumatic catheterization in anatomically demanding cerebral vessels (21). The stent-carrier module of the p64MW features an independently movable wire that can be placed 6 cm distal to the stent in order to provide increased stability during placement and preserve distal access after deployment. The potentially most significant improvement is its availability with a hydrophilic polymer coating that inhibits platelet adhesion and activation, and thus allows early reduction of DAPT or even single anti-platelet therapy (SAPT) (17, 22–26).

### Endovascular Procedure and Antiplatelet Regimen

All interventions were performed under general anesthesia using a biplane digital subtraction angiography system (Philips AlluraClarity, Best, The Netherlands). DAPT was performed as follows: each patient received a loading dose of 500 mg acetylsalicylic acid (ASA) together with either 180 mg ticagrelor, 30 mg prasugrel, or 300 mg clopidogrel 24 h prior to the procedure. DAPT was continued with 100 mg ASA daily and either 90 mg ticagrelor every 12 h (twice a day), 10 mg prasugrel daily, or 75 mg clopidogrel daily, for an average of 12 months. Clopidogrel as second anti-platelet drug was chosen only in patients who had already been treated with clopidogrel in an earlier occasion. Ticagrelor or prasugrel was chosen in patients who required DAPT for the first time, as platelet function testing is not routinely performed in all institutions and both agents are not associated with high-on-treatment platelet reactivity, as it is the case for clopidogrel (10). Overall, the majority of patients (20/32) received DAPT consisting of ASA and ticagrelor. DAPT with ASA and prasugrel or ASA and clopidogrel was administered in eight patients, four received prasugrel as second drug, and four received clopidogrel as second drug. In further four cases, decision was made for SAPT (ticagrelor in one or prasugrel three patients) based on a preexisting anticoagulation (2/4 patients) and a case of ASA intolerance, respectively. In the remaining case, SAPT with prasugrel was amended by celecoxib and dexamethasone in regard of a giant aneurysm with distinct mass effect. A bolus of 5,000 international units heparin was administered at the beginning of each procedure.

In all patients, triaxial endovascular access was established *via* the right common femoral artery using an 8 French introducer sheath (Terumo radifocus II, Leuven, Belgium). For supra-aortic extra-cranial access either the Neuron Max 088 (6F; Penumbra, Alameda, CA, USA) or the Cerebase (Cerenovus, Miami, FL, USA) were used. Either a 6F Sofia distal access catheter (115 cm; MicroVention, Aliso Viejo, CA, USA; *n* = 25) or a 6F Navien distal access catheter (Covidien Vascular Therapies, Mansfield, MA, USA) was used to enhance intracranial stability. Finally, the Headway 21 (MicroVention) microcatheter was used for device delivery in 20 cases, followed by the Rebar 18 (Medtronic, Covidien, USA) in nine cases, and the Prowler Select Plus (Codman Neurovascular, Raynham, MA., USA) in three cases.

### Procedure Assessment, Radiological and Clinical Follow-Up

After the implantation, the patency of the parent artery and covered side branches as well as FDS efficacy employing the O'Kelly–Marotta scale [OKM; (27)] were angiographically assessed in each case. The OKM grading scale defines the degree of residual aneurysm filling (A-D) and the angiographic opacification referring to the intra-aneurysmal stasis lasting up to the arterial, capillary, or venous phase. After the procedure, all patients were transferred to the intensive care unit (ICU) ensuring continuous monitoring of the patient's neurological status for at least 24 h. Cranial computed tomography (CCT) was performed 24 h after the intervention as a post-interventional standard imaging. Angiographic follow-up examinations were aimed at 3, 9, and 24 months after FDS implantation.

## Results

### Patients and Aneurysms

**Figure 1:**
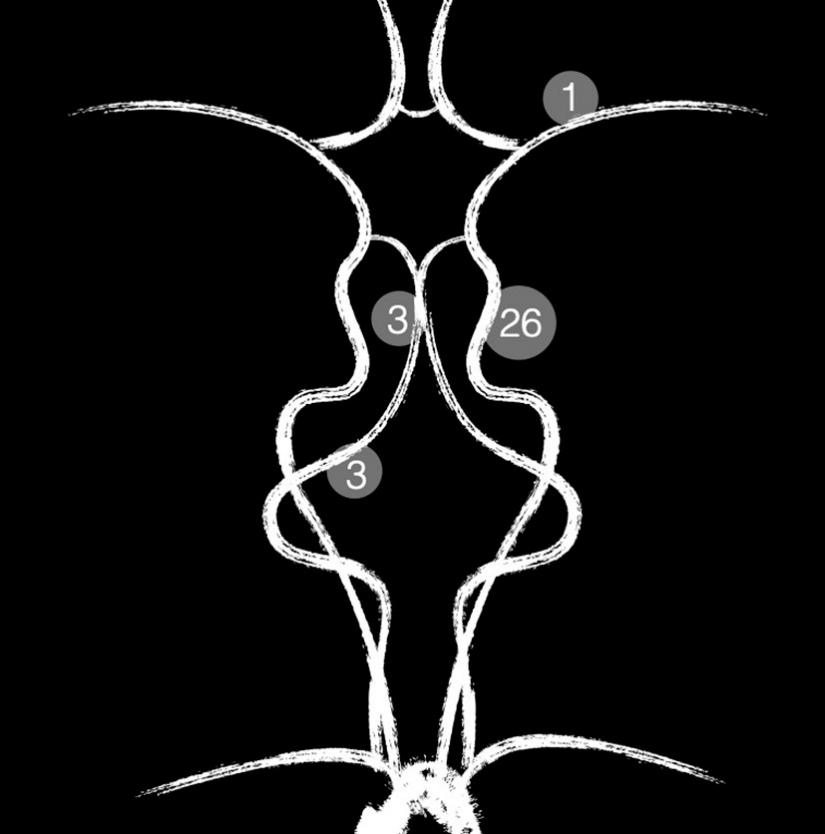
Summary of the anatomical distribution of the treated lesions.

Overall, 32 patients (22 female, 10 male) were treated with the p64MW-HPC. The average age at presentation was 57 years, ranging from 24 to 82 years. One patient had two adjacent aneurysms, which were treated in one session with a singular FDS. Twenty-eight lesions were saccular side-wall aneurysms. The remaining lesions were one blister aneurysm, one fusiform aneurysm, and two dissecting aneurysms. Another patient suffered from a high flow carotid-artery-cavernous-sinus-fistula (Barrow A). Figure 1 summarizes the anatomical distribution of the treated lesions.

### Treatments and Procedural Aspects

**Figure 2:**
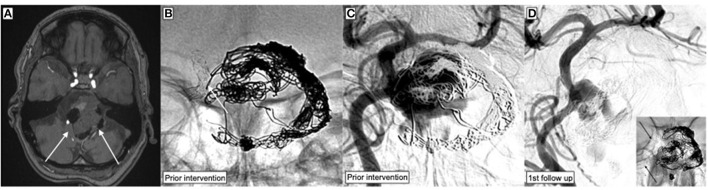
Endovascular treatment of a wide-necked, partially thrombosed giant aneurysm arising from the side wall of the basilar artery, which had been treated with stent-assisted coiling 6 years earlier but without any follow-up. The aneurysm relapsed and caused brain stem compression. **(A)** Time of flight angiography (TOF) shows the partially thrombosed basilar artery aneurysm (white arrows) compressing the brain stem. **(B)** Device radiography demonstrates the separation of the previously implanted Neuroform stents (white arrow) and substantial deformation of the coil package. **(C)** DSA image corresponding to the radiography in **(B)**. The gap between the stents corresponds to the broad neck of the aneurysm. The same projection was used for FDS implantation. **(D)** The p64MW-HPC was implanted within the Neuroform stents in order to bridge the gap between the separated devices and provide a scaffold for the formation of a neo-intima along the neck of the aneurysm.

**Figure 3:**
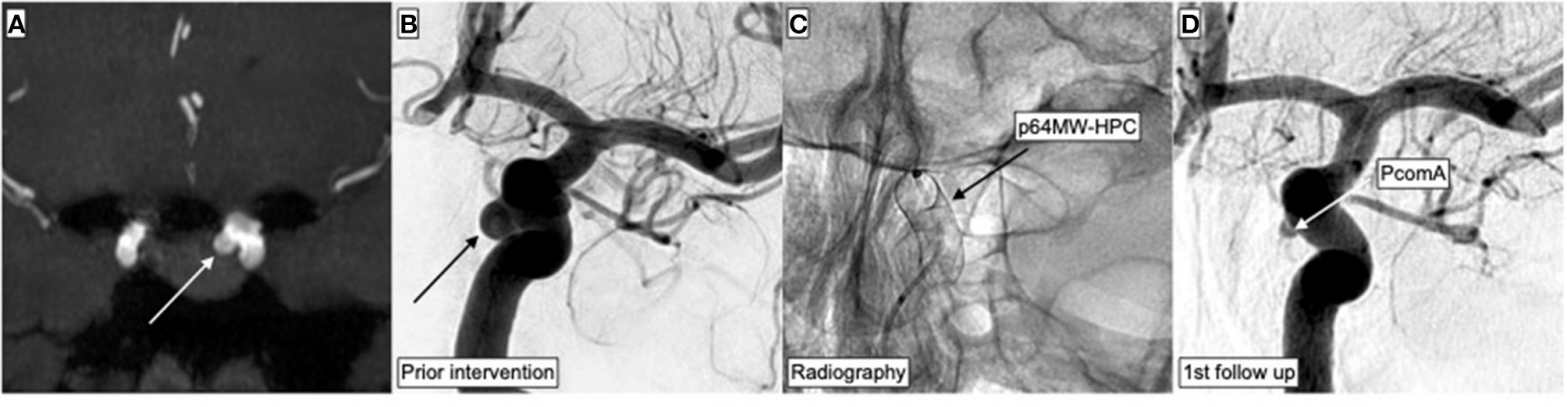
Endovascular treatment of an incidental aneurysm arising from the left-handed side ICA. MRI was performed as preoperative imaging for planning of pituitary adenoma surgery. The growth-hormone-secreting adenoma had caused acromegaly. After detection of the aneurysm, decision was made for preceding flow diversion in order to facilitate future transsphenoidal resection without increased risk for arterial bleeding. **(A)** TOF-angiography maximum intensity projection (MIP) demonstrates the 4.5-mm measuring ICA aneurysm and its anatomical relationships. **(B)** Corresponding DSA in posterior–anterior projection demonstrates the aneurysm (black arrow) prior to treatment. **(C)** Radiography immediately after implantation of the p64MW-HPC in a posterior–anterior projection, matching **(B,D)**. **(D)** First follow-up DSA after 3.5 months in posterior–anterior projection; the aneurysm is completely occluded.

In sum, 39 p64MW-HPC were successfully implanted. Detailed information about the procedural aspects are given in Table 2. The vast majority (28 patients; 87.5%) was treated with FD using the p64MW-HPC as primary and only endovascular implant. In four patients (12.5%), flow diversion was performed as a second step after initial embolization (plug and pipe). An example of flow diversion after coiling is shown in Figure 2, whereas Figure 3 demonstrates flow diversion as a sole strategy for an aneurysm associated with a growth-hormone secreting pituitary adenoma prior to transsphenoidal surgery.

**Table 2:** Procedural aspects.

Number of patients	*n* =32
Number of treated lesions	*n* = 33
Number of implanted devices	*n* = 39
Number of implanted p64MW(HPC) per patient	
1	26
2	5
3 or more	1
Adjunctive devices	6
Technical adverse events	*n* = 5
Twist of the FDS	3
Insufficient wall adaption	1
Device shortening	1
Periinterventional adverse events	*n* = 6
Delay in distal perfusion	3
Thrombus formation/ vessel occlusion	2
Extravasate	1
Clinical adverse events	*n* = 3
Transient stent occlusion	2
Delayed aneurysm rupture	1
Clinically manifest adverse events at last follow up	0

**Figure 4:**
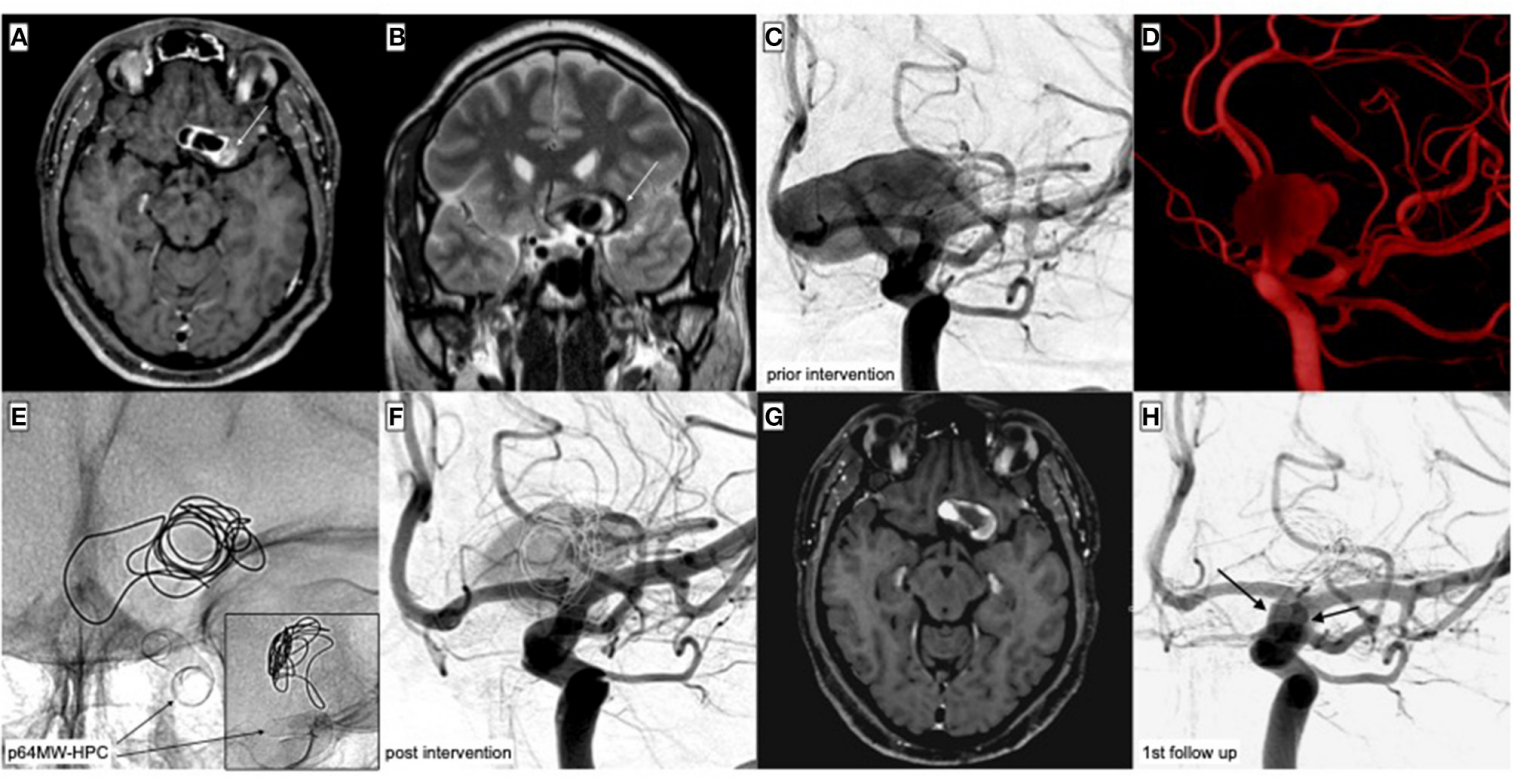
Treatment of a partially thrombosed, giant aneurysm of the C6 segment of the left-handed side ICA. The aneurysm had compressed the ipsilateral optic nerve and caused unilateral blindness. The cause for unilateral blindness had remained unclear for months—finally, MR imaging was performed and demonstrated the underlying pathology. The decision was made for flow diversion together with loose additional coiling, in order to further decrease intra-aneurysmal flow. **(A)** Contrast-enhanced, axial T1-weighted imaging shows the partially thrombosed (white arrow) giant aneurysm of the left-handed side ICA. **(B)** Coronal T2-weighted imaging demonstrates the multi-compartmental (white arrow) aneurysm and its significant space-occupying effect. **(C)** DSA in posterior–anterior projection shows the 26 × 17 mm perfused compartment of the aneurysm. **(D)** Reconstruction of the 3D rotational angiogram demonstrating the neck level of the giant aneurysm. **(E)** Radiography in posterior–anterior projection post implantation and coiling. The distal end of the p64MW-HPC was positioned in the C7 segment of the ICA, avoiding affection of the ICA-T. One coil was placed inside the aneurysm in order to further disrupt intra-aneurysmal flow. The image in the lower right corner displays the lateral projection after FDS implantation and supplementary coiling. **(F)** DSA immediately after implantation of the FDS. Note the already decreased volume of the residually perfused compartment. Compared to **(C)**, aneurysmal perfusion is markedly delayed. **(G)** Contrast-enhanced, axial T1-weighted imaging 5 weeks post procedure demonstrating the significantly decreased volume of the aneurysm together with progressive thrombosis of the peripheral compartments. **(H)** First angiographic follow-up in posterior–anterior projection 3 months post intervention. The aneurysm is sufficiently separated from the parent vessel (OKM C2).

Supplementary devices were required in six cases. In two patients who suffered from aneurysms >8 mm diameter, additional coiling of the aneurysm was performed aiming to promote intra-aneurysmal flow reduction and thrombus formation. Figure 4 illustrates an exemplary case—the treatment of a giant sidewall aneurysm with FDS and coiling. Another patient, who presented with a symptomatic high-flow carotid artery cavernous sinus fistula, was treated with flow diversion and transvenous coil embolization of the affected cavernous sinus compartment. The patient with the megadolicho-basilar artery was treated with FD and coil occlusion of the contralateral distal vertebral artery in order to prevent thromboembolism from the covered branch. In further two patients, FD was augmented with implantation of a second FDS within the previously implanted p64MW-HPC. One patient, who presented with a giant aneurysm arising from the cavernous ICA, received two additional PED2 shield, which were implanted as stent-in-stent construct. The second patient, who suffered from an incidental, para-ophthalmic aneurysm, received a p64MW-HPC that initially showed poor wall adaption and then shortened after catheterization with the microcatheter in order to promote wall approximation. A Silk Vista Baby (SVB; Balt, Montmorency, France) was subsequently implanted, which improved wall adaption and achieved an anatomically optimal result.

Delayed perfusion of covered branches occurred in two patients immediately post implantation. An intravenous bolus of body weight-adapted integrilin (= eptifibatid, GlaxoSmithKline, Dublin, Ireland) was given intravenously, which resolved the issue in all cases without further sequelae.

### Adverse Events

#### Technical Adverse Events

Purely technical adverse events in the early phase were observed in five cases (15.6%). Among them, twisting of the p64MW-HPC occurred in three patients and was related to distinct ICA tortuosity in all cases. The movable wire, however, allowed successful re-probation of the segment distal to the implanted FDS with some effort. Balloon angioplasty was performed subsequently, resulting in complete opening and wall adaption of the FDS in each of the cases. As described above, in a case of poor wall adaption and shortening, the technical issues were compensated by implantation of an additional low profile FDS (Silk Vista Baby). In a further patient, shortening of the distal end post implantation caused incomplete aneurysm coverage. A second p64MW-HPC was successfully implanted, resolving the issue completely. Clinical sequelae related to those technical obstacles were not observed in any case.

In one patient presenting with a PICA aneurysm, secondary dislocation of the FDS occurred after implantation in the V4 segment. Although the p64MW-HPC was initially well-positioned confirmed by digital subtraction angiography (DSA) immediately after deployment, the first angiographic follow-up revealed significant distal migration of the FDS. However, the device still covered the aneurysm neck, and the aneurysm was completely excluded from the intracranial circulation.

#### Clinical Adverse Events

Overall, clinical complications occurred in three patients (9.4%), two were of thromboembolic and one of hemorrhagic nature. Both thromboembolic complications occurred in patients who initially presented with a right-sided ICA aneurysm that was sufficiently treated with a singular p64MW-HPC. However, 1½ weeks after implantation, each of the patients presented with an acute onset, left-sided hemiparesis. Thrombus formation within the implanted devices causing impaired perfusion of the ICA territory was revealed by the immediately performed DSA. Both patients received integrilin as outlined above. The thrombus and patients' neurological deficits resolved completely. Both cases were related to abandoned anti-platelet medication. DAPT was re-initiated and then continued without further adverse events.

In one patient, delayed aneurysm rupture 2.5 weeks after FDS implantation for treatment of a right-sided cavernous ICA aneurysm caused a high-flow carotid artery cavernous sinus fistula (Barrow A). The patient had acutely developed headache, ipsilateral tinnitus, and exophthalmos. After confirmation of the diagnosis, the fistula was treated in two separate sessions. Firstly, two additional p64MW-HPC were implanted, aiming to reduce the volume of the AV-shunt. Complementarily, *via* a transfemoral venous approach, cavernous sinus coiling was performed, but only resulted in reduction, not occlusion of the shunt. In a second session, complete occlusion was achieved after coiling of a venous pouch *via* the ophthalmic vein. At the last clinical follow-up, the patient had recovered completely from the complication.

### Angiographic Follow-Up

#### Hemodynamic Effect Immediately After Implantation

Angiographic evaluation of the therapeutic effect was performed immediately after FDS implantation using the OKM grading scale. From a total of 32 assessed aneurysms, 20 (62.5%) revealed a distinctly delayed perfusion, corresponding to OKM grades A2–A3. In eight cases (25%) the aneurysm dome remained partially perfused (OKM grades B1–B3). A residual neck perfusion (OKM grades C1–C3) was observed in one case (3.1%). In the case of a fusiform dissecting aneurysm of the dominant vertebral artery, which was associated with an additional blister aneurysm, functional reconstitution of the lumen verum with separation of the pseudoaneurysm was achieved immediately after FDS implantation, corresponding to OKM grade D1. In sum, all but two aneurysms (93.75%) already showed a marked delay in perfusion (OKM grades A2–A3) or additional reduction of the opacified aneurysmal volume, corresponding to OKM grades B1–B3, C1–C3, and D1 immediately after treatment with the p64MW-HPC. Two aneurysms (6.25%) revealed no substantial residual opacification after implantation (OKM grades C3 and D1).

#### Hemodynamic Effect at the First Follow-Up Imaging

The first angiographic follow-up after a mean of 2.5 months was available for 23 lesions in 22 patients. Twelve of the 23 aneurysms (52.2%) revealed sufficient exclusion from the intracranial circulation, corresponding to OKM grades C1–C3 and D1. In six cases (26.1%), the aneurysm dome remained partially opacified (OKM grades B1–3), but showed a markedly reduced residual volume. Three lesions (13%) revealed a significantly delayed perfusion without changes in volume (OKM grades A2–A3). In conclusion, similar to the initial evaluation, all but two aneurysms (91.3%) showed a significant reduction of aneurysm perfusion (OKM grades A2–A3) or decreased, residually perfused volumes together with prolonged stasis of the contrast agent (OKM grades B1–B3, C1–C3, and D1). Thus, successful early hemodynamic remodeling, corresponding to OKM grades C1–C3 and D1, was achieved in 52.2% of the lesions at the first follow-up after 2.5 months. Only 2 of the 23 aneurysms (8.7%) remained morphologically unaltered (OKM A1).

#### Hemodynamic Effect at the Second Follow-Up Imaging

A second imaging follow-up, on average 6 months post procedure, was performed in 13 of the 22 patients (=14 aneurysms), who had received the first follow-up. Eight of the 14 aneurysms (57.1%) were completely or subtotally excluded from the intracranial circulation (OKM grades C1–C3 and D1). Residual perfusion (OKM grades B1–B3) was observed in four aneurysms (28.6%). Two aneurysms (14.3%) showed a significant delay of contrast outwash (OKM A3). There was no case of unaltered aneurysm perfusion (OKM A1).

#### Overall Hemodynamic Outcome at the Last Available Follow-Up Imaging

Considering the last available imaging follow-up of all patients, i.e., five patients after ~7.5 months, seven patients (eight aneurysms) after 6 months and 10 patients after 2.5 months, 15 aneurysms (65.2%) were already functionally separated from the intracranial circulation (OKM grades C1–C3 and D1). A significant decrease of the residually perfused aneurysm volumes (OKM grades B1–B3) occurred in six cases (26.1%). Two aneurysms (8.7%) showed no reduction in perfused volume, but significantly prolonged opacification (OKM grade A3). Overall, reduction of aneurysm influx and morphological improvement was achieved in all aneurysms treated with the p64MW-HPC. No case of unaltered aneurysm perfusion (OKM A1) was observed.

Overall, in-stent stenosis, probably representing endothelialized mural thrombus, as reported recently (28), occurred in eight patients. Six cases were mild, two were moderate, and none was hemodynamically significant.

## Discussion

Our retrospective three-center study reports the efficacy of aneurysm treatments employing the p64MW-HPC, a newly developed low-profile FDS with improved hemocompatibility, together with complications, clinical aspects, technical pitfalls, and their management. Besides first clinical evidence for the safety and feasibility of the novel FDS for treatment of cerebral aneurysms in a German patient collective, our study addressed the question of FD efficacy in the presence of the HPC surface modification, and provided case-based clinical evidence for the necessity of DAPT in the early post-interventional phase despite the use of HPC-modified FDS.

In general, our findings demonstrated a good efficacy of the p64MW-HPC for aneurysms arising from proximal cerebral vessels, i.e., the intradural internal carotid artery, the M1 segment of the middle cerebral artery, and the proximal posterior circulation including the basilar and vertebral arteries. After ~6 months, 65% of the aneurysms treated with the p64MW-HPC were sufficiently isolated from the parent vessel. These findings are largely in accordance with earlier studies investigating the efficacy of nowadays well-established bare-metal FDS, for example, the first-generation “classic” p64 or the Pipeline Embolization Device (PED), which demonstrated occlusion rates for proximally located aneurysms between 58.3 and 73.6%, 3–6 months post implantation (29–32).

However, recent studies on the efficacy of the third-generation PED with Shield Technology, a phosphorycholine-based surface coating that mimics the red blood cell's outer membrane and significantly reduces platelet activation, reported even higher rates of early occlusions in up to 79.7% after 6 months (33–35). Considering the central role of platelets for the induction of neo-endothelialization (11), it appears somewhat conflicting that application of a platelet-inhibitory surface modification on a FDS is associated with enhanced occlusion rates (36). This dissonance is further contrasted by the fact that the hydrophilically coated p64MW, which is composed of a more densely woven and hemodynamically impactful mesh than the PED, achieved an inferior rate of early occlusions in our patient collective. The discrepant occlusion rates (65% vs. almost 80%) may simply reflect a greater hemodynamic complexity in the subset of aneurysms, which were treated with the p64MW-HPC (37). Aside from that, the anti-thrombogenic surface modifications either based on phosphorylcholine (Medtronic: Shield Technology) or hydrophilic glycan-containing polymers (Phenox: HPC) may interact differently with the circulating blood cells and the migratory cells from the vessel wall that are required for the formation of a neo-intima (9, 10), and hence, influence its progress in a divergent way. Indeed, an experimental study compared the uncoated with the coated PED and revealed faster neo-endothelialization together with decreased thrombus formation for the PED with Shield technology (38), supporting this hypothesis. Unfortunately, comparable investigations on the p64MW-HPC and its bare metal counterpart are lacking. As a consequence, further investigations are needed to better understand the impact of the distinct anti-thrombotic coatings on the process of vascular healing after FDS implantation (36, 39).

Moreover, the anti-platelet medication in our study differed considerably from the standard DAPT regimen in the aforementioned PED Shield studies, which comprised ASA and clopidogrel. High on-treatment platelet reactivity occurs with clopidogrel in up to 50% of patients (40) and is associated with thromboembolic complications in neurovascular stenting (41). As current methods of platelet function testing are not sufficiently reliable to predict treatment efficacy of DAPT, the clinical value of platelet function testing in FD remains doubtful (10, 42, 43). As a consequence, either ticagrelor or prasugrel was predominantly administered as a second anti-platelet drug in our cohort, in order to avoid the pitfalls associated with clopidogrel. Large-scale clinical trials demonstrated superiority of ticagrelor and prasugrel over clopidogrel regarding the prevention of ischemic complications without excess risk of hemorrhage (44). However, neo-endothelialization of a FDS seems to depend on the preceding formation of thrombus along its surface to some extent, and *vice versa*, excessive DAPT delays the process of arterial healing (36, 38). Hence, using the more potent anti-aggregants ticagrelor or prasugrel for DAPT in our patients may have contributed to prolonged vascular remodeling and, thus, decelerated aneurysm occlusion. Aside from platelet inhibition, circulating anti-aggregants impact the physiology of the endothelium directly *via* the P2Y12 receptor and through paracrine mechanisms that involve other circulating blood cells (45, 46). A growing number of *ex vivo* and clinical studies in this regard indicate that the currently used P2Y12-receptor antagonists, i.e., clopidogrel, prasugrel, and ticagrelor, have a very different effect on injury-repair process that immediately begins after stenting (47). The latter anti-platelet drug reduces neo-intimal hyperplasia together with the local inflammatory response and excessive proliferation of smooth muscle cell proliferation while preserving re-endothelialization (48). Schnorbus et al. compared the three P2Y12 anti-aggregants and postulated that prasugrel is associated with improved endothelial function, stronger platelet inhibition, and a lower inflammatory response compared to ticagrelor and clopidogrel (49). Other authors claimed the superiority of ticagrelor over prasugrel and clopidogrel regarding the extent of platelet inhibition and endothelial function (44).

In summary, the P2Y12 inhibitors not only inhibit platelet activation to very different extents, but also exert a number of drug-specific pleiotropic effects that influence vascular physiology after stenting. However, in particular, which P2Y12 antagonist provides the best pharmaceutical profile for flow diversion remains to be elucidated, and prospective studies in this regard are urgent. From the current perspective, a certain degree of customization of anti-platelet medication, accounting for the type and surface modification of the respective implant in the context of the patient's hemostaseologic condition and potentially required level of care, should be considered to achieve an optimal outcome. In our patient collective, for example, two ischemic complications manifested as a result of suddenly abandoned anti-platelet medication within the 3rd week post intervention. The respective patients required a moderate level of care related to their preexisting comorbidities and were supported in day care centers. In retrospect, DAPT, consisting of ASA and ticagrelor in both cases, was given incompletely after returning to day care, resulting in acute in-stent thrombosis. Both cases were treated sufficiently as described above and the patients recovered well from the complications. However, ticagrelor, although having a low rate of hypo-response, has a very short duration of action, requires the intake of two pills every 12 h and skipping just one pill can cause device thrombosis (10). Therefore, prasugrel, exhibiting a longer duration of action and requiring only 1 pill every 24 h, may have been the more appropriate anti-aggregant in this situation.

The occurrence of complications in the especially sensitive, early post-interventional phase is well-known and our observations in this regard are in accordance with previous studies (50, 51). Both thromboembolic and hemorrhagic events are comparatively rare, but oftentimes manifest within 6 weeks post FDS-implantation (52). However, the complications in our patients were treated with good outcomes and no patient suffered from severe, permanent device-related morbidity. The rate of clinical complications in our preliminary study on the p64MW-HPC is in line with or below the complication rates of earlier reports (20).

The five technical adverse events in our study were dominated by twisting of the p64MW-HPC in highly tortuous cavernous or ophthalmic ICA segments, which were successfully resolved by balloon angioplasty. In those cases, the movable wire of the stent-carrier module maintained distal endovascular access and facilitated the introduction of the balloons markedly. However, recognition and immediate elimination of twisting or device collapse are paramount, as they functionally equal the occlusion of the vessel (20). Secondly, foreshortening or migration of the p64MW-HPC occurred as further technical issues. In our study, peri-interventional shortening was sufficiently compensated by implantation of a second low-profile FDS, and the distal migration did not require a further intervention. Nevertheless, both phenomena can culminate in fatal SAH, for example, if the distal end of the FDS dislocates into the aneurysm sac, directing blood flow toward the aneurysm wall, or major ischemic stroke, if the device migrates distally and obstructs a downstream bifurcation (53). Migration and foreshortening are related to suboptimal wall adaption and the specific low-porosity closed cell design of FDS (54). Hence, their occurrence is limitable by appropriate sizing and optimal placement. However, as they also may manifest several months after implantation, regular follow-ups during the 1st year are important (54).

Our study suffers from a number of limitations. Firstly, it is a retrospective analysis of a prospectively maintained database. Related to the novelty of the device, only a small number of patients with limited numbers of follow-up examinations were available. The lack of routinely performed platelet-function testing, in addition, limits the assessment of the efficacy of the administered anti-platelet regimen and its potential impact on the aneurysm occlusion rate. Furthermore, intraluminal imaging, for example optical coherence tomography, which is well-suited to assess the process of neo-endothelialization, was not performed (55).

## Conclusion

This study highlights the safety and feasibility of the novel p64MW-HPC for the treatment of intracranial aneurysms arising from proximal segments of the anterior and posterior intracranial circulation. The early follow-up results indicate good early occlusion rates, which appear comparable to bare-metal FDS. The compatibility with small 0.021” microcatheters limits the necessity for potentially traumatic catheterization maneuvers and facilitates FDS implantation in tortuous, elongated vessels. However, further studies and long-term data are required in order to assess the efficacy of the p64MW with HPC surface modification. A comparative prospective study between the uncoated p64MW and the p64MW-HPC employing intraluminal imaging is wanted.

## Data Availability Statement

The original contributions presented in the study are included in the article/supplementary material, further inquiries can be directed to the corresponding author/s.

## Ethics Statement

The studies involving human participants were reviewed and approved by Ethikkommission des Universitätsklinikums Leipzig. The patients/participants or his or her legal representative provided their written informed consent to participate in this study.

## Author Contributions

HW was responsible for data acquisition, performed statistical review, and wrote the paper. M-SS was responsible for data acquisition, wrote the paper, and performed image analysis. CS performed interventions and follow-up imaging analysis. DM and JT performed interventions, follow-up imaging, and vascular analysis. WH reviewed and drafted the paper. AD performed image analysis and reviewed the paper. JM and NB performed follow-up imaging and analysis. KH wrote and reviewed the paper. UQ performed interventions. SS designed the study, wrote the paper, and performed interventions. All authors contributed to the article and approved the submitted version.

## Conflict of Interest

UQ has proctoring and consultancy agreements with phenox and Balt Germany. SS has proctoring and consultancy agreements with phenox and Balt international. The remaining authors declare that the research was conducted in the absence of any commercial or financial relationships that could be construed as a potential conflict of interest.

## Publisher's Note

All claims expressed in this article are solely those of the authors and do not necessarily represent those of their affiliated organizations, or those of the publisher, the editors and the reviewers. Any product that may be evaluated in this article, or claim that may be made by its manufacturer, is not guaranteed or endorsed by the publisher.
